# Insights Regarding the Role of Inflammasomes in Leukemia: What Do We Know?

**DOI:** 10.1155/2023/5584492

**Published:** 2023-08-04

**Authors:** Fabíola Silva Alves-Hanna, Juniel Assis Crespo-Neto, Glenda Menezes Nogueira, Daniele Sá Pereira, Amanda Barros Lima, Thaís Lohana Pereira Ribeiro, Vitória Giovanna Rodrigues Santos, Joey Ramone Ferreira Fonseca, Fábio Magalhães-Gama, Aya Sadahiro, Allyson Guimarães Costa

**Affiliations:** ^1^Programa de Pós-Graduação em Imunologia Básica e Aplicada, Instituto de Ciências Biológicas, Universidade Federal do Amazonas (UFAM), Manaus, AM, Brazil; ^2^Diretoria de Ensino e Pesquisa, Fundação Hospitalar de Hematologia e Hemoterapia do Amazonas (HEMOAM), Manaus, AM, Brazil; ^3^Programa de Pós-Graduação em Ciências Aplicadas à Hematologia, Universidade do Estado do Amazonas (UEA), Manaus, AM, Brazil; ^4^Programa de Pós-Graduação em Ciências da Saúde, Instituto René Rachou, Fundação Oswaldo Cruz (FIOCRUZ-Minas), Belo Horizonte, MG, Brazil; ^5^Escola de Enfermagem de Manaus, Universidade Federal do Amazonas (UFAM), Manaus, AM, Brazil

## Abstract

Inflammation is a physiological mechanism of the immune response and has an important role in maintaining the hematopoietic cell niche in the bone marrow. During this process, the participation of molecules produced by innate immunity cells in response to a variety of pathogen-associated molecular patterns and damage-associated molecular patterns is observed. However, chronic inflammation is intrinsically associated with leukemogenesis, as it induces DNA damage in hematopoietic stem cells and contributes to the creation of the preleukemic clone. Several factors influence the malignant transformation within the hematopoietic microenvironment, with inflammasomes having a crucial role in this process, in addition to acting in the regulation of hematopoiesis and its homeostasis. Inflammasomes are intracellular multimeric complexes responsible for the maturation and secretion of the proinflammatory cytokines interleukin-1*β* and interleukin-18 and the cell death process via pyroptosis. Therefore, dysregulation of the activation of these complexes may be a factor in triggering several diseases, including leukemias, and this has been the subject of several studies in the area. In this review, we summarized the current knowledge on the relationship between inflammation and leukemogenesis, in particular, the role of inflammasomes in different types of leukemias, and we describe the potential therapeutic targets directed at inflammasomes in the leukemic context.

## 1. Introduction

Leukemias are a heterogeneous group of neoplasms with a broad clinical spectrum that is characterized by the impairment of a hematopoietic stem cell (HSC) and the blockage of differentiation at various stages of the maturation process, which are divided into acute myeloblastic leukemia (AML), chronic myeloblastic leukemia (CML), acute lymphoblastic leukemia (ALL), and chronic lymphoblastic leukemia (CLL) [1]. The incidence rates for leukemias differ between subgroups. In chronic leukemias, there is a trend toward an increase in cases in adults; however, in ALL, pediatric patients are predominant (<15 years old), with a possible appearance after 50 years of age. On the other hand, the other subtypes (CLL, CML, and AML) occur more frequently in adults over 65 years of age [2].

Chronic inflammation has been described as a key factor in the development of leukemia and other hematologic malignancies since it limits the proliferation of normal HSCs and contributes to the growth of cells with mutations [3]. Thus, a number of studies state that several intrinsic and extrinsic factors influence the malignant transformation within the inflammatory microenvironment. Intrinsic factors mainly encompass genetic changes in cell signaling pathways that regulate inflammation (such as NF-*κ*B), while extrinsic factors include the inflammatory pathways activated by the bone marrow microenvironment and include chemokines, cytokines, and adhesion molecules [4].

In recent years, studies have highlighted the important role of inflammasomes in hematopoietic homeostasis via the regulation of processes of differentiation and senescence of HSCs [5]. Inflammasomes are intracellular multimeric complexes formed during the systemic immune response and inflammation, whose main functions are the secretion of cytokines interleukin-1*β* (IL-1*β*) and interleukin-18 (IL-18), in addition to the process of inducing cell death via pyroptosis via the activation of caspase-1. IL-1*β* secretion is also responsible for stimulating other inflammatory cytokines, such as IL-1*α*, tumor necrosis factor (TNF)-*α*, and IL-6, thus amplifying the inflammatory cascade [6].

Chronic exposure of HSCs to IL-1*β* can lead to a deterioration of the hematopoietic niche, exhaustion of HSCs, and failures in regeneration [7]. In leukemias, there is an overproduction of IL-1*β* cytokines and other inflammatory cytokines, such as TNF-*α* and IL-6 [8, 9], which may be associated with tumor progression. Currently, the role of inflammasomes is well understood in some solid tumors, such as colon cancer [10]; however, in leukemia, its role is still quite controversial. Because of this, we performed a literature review and summarized the main findings regarding inflammasomes in leukemias to serve as a basis for directing therapeutic targets based on inflammasome components and thus help patients with leukemia.

## 2. Inflammation as a Trigger for Leukemia

Inflammation is defined as a protective immune response to infection and tissue damage mediated by the proinflammatory action of effector cells, cytokines, and chemokines, which orchestrate a systemic and/or local response [11]. HSCs are known as key cells in systemic inflammatory responses that are capable of integrating inflammatory stimuli into cellular responses and establishing a demand-adapted axis between peripheral stress and hematopoietic response in the bone marrow [12]. Although it is important for the maintenance of the hematopoietic niche, inflammation can contribute to the emergence of mutations and confer selective advantages to certain clones due to prolonged exposure [13]. The selective pressure imposed by inflammation on the pool of HSCs can induce genetic mutations and select inflammation-adapted mutant clones that can potentially progress to a leukemic condition [4, 12].

**Figure 1:**
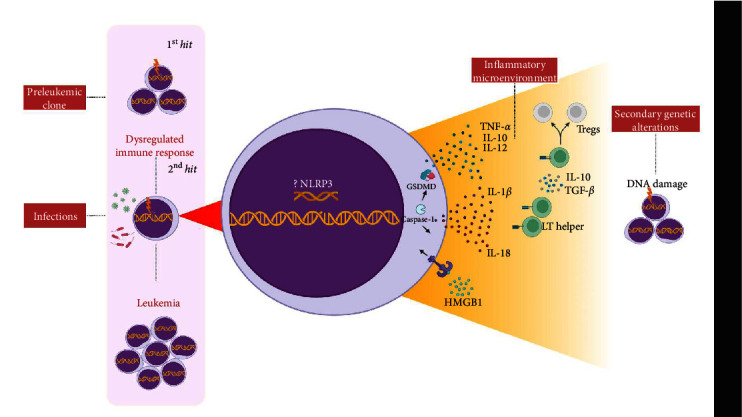
Inflammation-based model of leukemia development. A genetic alteration produced in the uterus would be responsible for the creation of a leukemic clone. After birth, due to the low stimulation of the immune system, a dysregulated immune response to common pathogens would occur, culminating in an exacerbated inflammatory response. Inflammasomes stand out as important mediators of inflammation in innate immunity through the recognition of PAMPs and DAMPs, which leads to the maturation and secretion of proinflammatory cytokines IL-1*β* and IL-18, and through the cell death process via pyroptosis. The cytokine IL-1*β* has pleiotropic functions in the tumor and can be secreted by both leukemic and stromal cells. It is responsible for the recruitment of myeloid-derived suppressor cells (MDSCs) that secrete IL-10 and TGF-*β* and induce the differentiation of TCD4+ lymphocytes into Tregs, thus promoting immunosuppression. In addition, binding to the IL1R receptor promotes a positive loop of autocrine/paracrine secretion of this cytokine in leukemic cells and, through activation of the NF-*κ*B pathway, this leads to the transcription of other inflammatory mediators, such as TNF-*α*, IL-10, and IL-12, that are secreted into the TME. Together, these mechanisms are responsible for inducing mitochondrial dysfunction, oxidative stress, and persistent DNA damage, which lead to the acquisition of secondary genetic alterations that contribute to the development of leukemia. Abbreviations: DAMPs, damage-associated molecular patterns; DNA, deoxyribonucleic acid; IL-10, interleukin-10; IL-12, interleukin-12; IL-18, interleukin-18; IL-1*β*, interleukin-1*β*; PAMPs, pathogen-associated molecular pattern molecules (PAMPs); TCD4+, lymphocyte CD34+; TGF*β*, transforming growth factor *β*; TME, tumor microenvironment; TNF-*α*, tumor necrosis factor-*α*; Tregs, regulatory T cells; ?, Influence on the known.

Taking this into account, Greaves [14–16] describes that the exacerbated immune response to common pathogens in early childhood may be responsible for inducing genetic alterations that could lead to the onset of leukemia. The so-called “hygienic hypothesis” is based on the direct relationship between the development of the immune system and exposure to infections in the pathogenesis of the disease, and this is demonstrated in Figure 1. Greaves [14–16] describes the model in two *hits*; the first *hit* is the acquisition of a prenatal genetic alteration (e.g., *ETV6::RUNX1* or hyperdiploid), which is a developmental error common that can give rise to a preleukemic cell. From the low stimulation of the immune system in early childhood or deficiency of “immune priming,” the infection by common pathogens would subsequently be responsible for triggering an exacerbated immune response and would culminate in the induction of secondary genetic alterations.

According to previous studies, we believe that sustained inflammation may play an important role in preventing immune surveillance of leukemic cells and promoting genomic instability [17]. Thus, the preleukemic clone [14–16] could cause alterations in the cytoskeleton, deregulate the expression of adhesion molecules and modify the cell migration by compromising the CXCL12/CXCR4 axis, thereby promoting its migration to extramedullary organs [12, 18].

During the intense immune response caused by an infection, for example, the proinflammatory cytokines IL-6, TNF-*α*, and IL-1*β* cooperate with MSCs to create a favorable niche for preleukemic cells by which there is the secretion of CXCR2 ligands that attract preleukemic cells (e.g., *ETV6::RUNX1+*) activin (ACTA)-dependents [18, 19]. In addition, after infection or injury, the cytokine IL-1*β* is produced in bone marrow and promotes myeloid differentiation through activation of the NF-*κ*B pathway that results in the expansion of hematopoietic stem and progenitor cells (HSPCs) [20]. However, chronic exposure to IL-1*β* significantly impairs self-renewal and the ability to differentiate HSPCs [21] and causes cellular stress by inducing a state of chronic oxidative stress with elevated levels of reactive oxygen species (ROS) and, in a positive looping process, amplifies the inflammatory response through the activation of NLRP3 via recognition of damage-associated molecular patterns (DAMPs) (e.g., adenosine triphosphate (ATP) and HMGB1) [22], which creates a high-risk microenvironment for inducing genetic alterations in hematopoietic cells [23].

The action of the cytokine IL-1*β* in the tumor microenvironment (TME) is mediated by the activation of inflammasome complexes. In general, in leukemias, IL-1*β* has been associated with (i) increased proliferation of leukemic cells [24–28] and (ii) recruitment of myeloid-derived suppressor cells (MDSCs) to the TME through the upregulation of the NF-*κ*B pathway, which promotes immunosuppression and favors the survival of the leukemic clone [29, 30]. MDSCs are responsible for the secretion of IL-10 and TGF-*β*, which contribute to the expansion of regulatory T lymphocytes (Tregs) in leukemias [31] and which may support the growth and survival of preleukemic cells through the release of cytokines, including TGF-*β* [32].

It is important to remember that leukemic cells can also secrete substances that contribute to clone survival. In a mouse model of the preleukemic disorder, dysregulated MSCs were able to release DAMPs (S100A8/9) that induced mitochondrial dysfunction, oxidative stress, and DNA damage in HSCs via paracrine activation of p53 and promoted malignant transformation [33]. In B-ALL, leukemic cells can produce and secrete inflammatory mediators, including TNF-*α*, IL-1*β*, IL-10, and IL-12 [34].

## 3. Inflammasomes

Inflammasomes are oligomeric protein complexes that form in the cytosol after the detection of pathogen-associated molecular patterns (PAMPs) and DAMPs. Although there are fundamental differences between stimulus-dependent inflammasomes, in general, they have the main function of recruiting the inactive zymogen procaspase-1, which after activation, will be responsible for the maturation and secretion of the proinflammatory cytokines IL-1*β* and IL-18, in addition to inducing the process of cell death via pyroptosis [35].

These complexes are formed by cytosolic sensors, an adapter component, and an effector component, such as caspase-1, -4, and -5 in humans and caspase-11 in murines. Sensor components, which inflammasomes are often named after, detect PAMPs and DAMPs and recruit adapters, which in turn recruit and activate caspases. The assembly of the inflammasome platform is a critical and well-organized process that involves several main parts, such as the sensors that recognize the activation signals and adapter molecules; the most common being the ASC (apoptosis-associated speck-like protein containing a CARD) and the effector molecule caspase [36].

Canonical inflammasomes are formed upon activation of two families of sensor molecules: NOD-like receptors (NLRs) and AIM2-like receptors. The human genome encodes 22 cytosolic proteins belonging to the NLR family, but only NLRP1, NLRP3, NLRP6, NLRP7, NLRP12, and the NAIP/NLRC4 complex can assemble their respective inflammasomes [37]. AIM2 (absent in Melanoma 2) and IFI16 (Interferon Gamma Inducible Protein 16) belong to the PYHIN family (PYD-like and HIN domain-containing proteins) [38–40].

The NLRs (except NLRP1) have a C-terminal domain that is rich in leucine repeats, which are responsible for recognizing a ligand, similar to the leucine-rich domain of Toll-like receptors (TLRs). In addition, they have a central nucleotide-binding domain (NACHT or NBD) that is responsible for the oligomerization of the receptor after activation, and a PYD effector domain in the N-terminal portion that triggers the effector function of the receptor by recruiting proteins to form signaling complexes [41].

NLRP1 (NLRs, pyrin-domain-containing proteins 1) is formed by an internal FIIND domain (function-to-find domain) and a CARD domain in the C-terminal region. NLRP1 is related to an inflammasome-forming PRR called CARD8 (caspase activation and recruitment domain-containing protein 8), which only has a predicted N-terminal∼160-amino-acid-long unstructured region followed by a FIIND and a CARD. Both NLRP1 and CARD8 undergo autoproteolysis at the C-terminal end of their ZU5 domains, generating N- and C-terminal fragments that remain noncovalently associated [42]. NLRC4 (NLR family CARD domain-containing protein 4) has a CARD domain at the N-terminal [38, 43], while the AIM2 and IFI16 sensors have an N-terminal PYD effector domain and a C-terminal HIN200 (hematopoietic interferon-inducible nuclear antigen with 200 amino-acid repeats) domain, which are responsible for recognizing the ligand [44, 45].

The ASC adapter protein is constituted by the PYD and CARD domains and acts in the recruitment of procaspase-1 through homotypic interactions of the CARD–CARD domains. The interaction between the procaspases results in the formation of the complex that induces its activation by autoproteolysis [46–50]. Classically, canonical inflammasome activation is initiated by two types of signals and is regulated at the transcriptional and post-translational levels. “Signal 1” is the initiation signal and is associated with the activation of the TLR/NF-*κ*B pathway or mitochondria-derived ROS that activate the TLR4/MyD88 signaling pathway. “Signal 2” can be induced by various stimuli, including PAMPs, DAMPs, ATP, and uric acid crystals [5].

After the formation of the complex, oligomerization of procaspase-1 proteins induces their autoproteolytic cleavage into activated caspase-1. When activated, caspase-1 (p20/p10 subunits) cleaves the inactive precursor forms of pro-IL-1*β* and pro-IL-18, leading to the maturation of the cytokines IL-1*β* and IL-18. In addition, the activation of caspase-1 can also cause cell death via pyroptosis through the activation of the gasdermin-D protein that is deposited in the cell membrane. This process is characterized by cell swelling, loss of plasma membrane integrity, and the release of inflammatory mediators due to the formation of pores in the membrane [51, 52].

**Figure 2:**
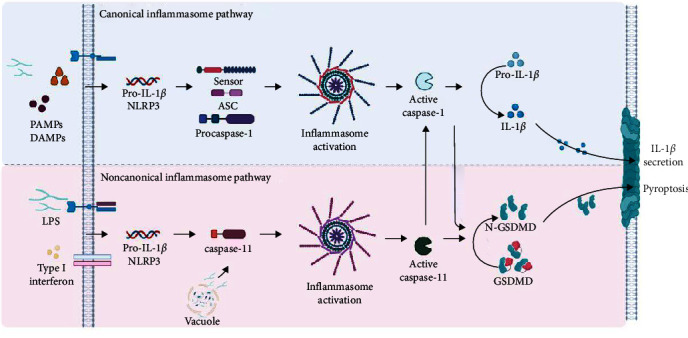
Overview of inflammasome activation pathways. After recognition of PAMPs or DAMPs by the sensors, oligomerization of the complex occurs, which induces the cleavage of procaspase-1 into activated caspase-1. (a) In the canonical pathway, activation requires priming by a Toll-like receptor (TLR) ligand (e.g., LPS)—mediated by MyD88 to induce the expression of pro-IL-1b and NLRP3. Pro-IL-18 is expressed constitutively in the cell. Caspase-1 activation cleaves the pro-IL-1*β* inactive precursor forms, leading to the maturation and secretion to the extracellular environment. In addition, gasdermin-D cleavage also occurs, which is deposited in the cell membrane, leading to the formation of pores and causing cell death via pyroptosis. (b) In the noncanonical pathway, extracellular LPS induces the expression of pro-IL-1b and NLRP3 via the TLR4-MyD88-dependent pathway and type I interferon via the TLR4-TRIF-dependent pathway. Type I interferon provides a feedback loop and activates IFNAR to induce caspase-11 expression. Cytosolic Gram-negative bacteria deliver LPS into the cytosol when they escape the vacuole. Caspase-11 is activated following its binding to cytosolic LPS, then drives pyroptosis and activation of the noncanonical NLRP3 inflammasome Abbreviations: DAMPs, damage-associated molecular patterns; GSDMD, gasdermin-D; LPS, lipopolysaccharide; N-GSDMD, amino-terminal cell death domain; PAMPs, pathogen-associated molecular pattern molecules; TLR, Toll-like receptor; MyD88, myeloid differentiation primary response 88; IFNAR, type I interferon receptor.

Although the final common pathway of canonical inflammasome activation involves the recruitment of caspase-1 in response to multiple PAMPs and DAMPs, the noncanonical inflammasome signals independently of caspase-1. In the noncanonical pathway, the sensor directly recognizes the intracellular LPS of Gram-negative bacteria through the CARD domain of caspases-4 and -5 (in humans) and caspase-11 (in mice). However, there is still a lack of convincing evidence regarding the involvement of these caspases in the process of maturation and cleavage of pro-IL-1*β* and pro-IL-18 cytokines, this being a specific function of caspase-1. Therefore, in this pathway, only gasdermin-D cleavage is observed, which is sufficient to promote cell lysis and activate the canonical inflammasome pathway [36]. In Figure 2, we show, in a summarized form, the inflammasome activation pathways.

## 4. The Role of Inflammasomes in the Regulation of Hematopoiesis

Hematopoietic cells are hierarchically organized by a pool of quiescent and pluripotent stem cells that are capable of self-renewal and generation of mature blood cells throughout life [53]. Studies indicate that the inflammasome is involved in different stages of hematopoiesis, and several types of its components have been shown to contribute to the maintenance and differentiation of HSPCs. Both up- and downregulation of inflammasome proteins can lead to physiological changes in homeostasis, suggesting that their activation may be necessary to carefully preserve hematopoiesis [5].

Master et al. [54] discovered that activation of the NLRP1*α* inflammasome in murines induced a lethal systemic inflammatory process triggered by pyroptosis in HSPCs. Interestingly, this causes prolonged cytopenia, bone marrow hypoplasia, and immunosuppression during periods of hematopoietic stress induced by chemotherapy or lymphocytic choriomeningitis virus infection, demonstrating that NLRP1 acts as a cellular sentinel to alert caspase-1 to hematopoietic and infectious stress [54]. On the other hand, the NLRP1*α* also plays a physiological role in HSPCs and leads to myeloid differentiation through the transcription factor GATA-1, and its deletion is responsible for the decrease in the myeloid lineage and the increase in the erythroid lineage [55]. These findings demonstrate a dual role of the NLRP1 inflammasome in the regulation of hematopoiesis and may be a potential target for the study of the development of hematologic malignancies and strategies for treating infection-induced cytopenias.

Under the effects of radiation, the activation of the AIM2 inflammasome is responsible for causing the death of HSPCs and medullary aplasia in mice. Hu et al. [56] observed that AIM2-deficient mice do not suffer from irradiation-induced hematopoietic failure, as AIM2 recognizes double-stranded DNA and mediates cell death in response to radiation-induced DNA damage. Furthermore, It has been proposed that inhibiting AIM2 inflammasome-mediated pyroptosis may be a strategy for preventing radiation-induced injuries as in radiotherapy [57]. In addition, exposure to low-dose ionizing radiation *in vitro* and *in vivo* is responsible for NLRP3 activation in THP-1 cells and the elevation of ROS levels [58].

Metabolic activity has been described as a critical factor regulating stem cell proliferation and differentiation. In addition, NLRP3 inflammasome-mediated IL-1*β* signaling in macrophages drives HSPC production in response to metabolic activity in a zebrafish model [59]. Linz et al. [60] demonstrated the positive effects of inflammasomes in hematopoiesis. NLRP12 impacts hematopoietic recovery by suppressing TNF signaling *in vivo* during emergency hematopoiesis induced by the combination of radiation exposure and thermal injury. The upregulation of NLRP12 functionally abolishes TNF-induced HSPCs apoptosis; however, when the *NLRP12* gene is deleted, there is HSPC apoptosis as well as defective peripheral immune reconstitution. Thus, myelopoiesis and immune cell reconstitution are accelerated by the overexpression of NLRP12 [60]. We, therefore, hypothesize that NLRP12 may serve as a potential inducer of hematopoiesis in transplant models.

The combination of physiology and pathological changes commonly occurs in the hematopoietic system and ultimately forms the basis of various types of hematological malignancies [61]. These findings demonstrate an important role for the inflammasome in the regulation of hematopoiesis and point to the need for studies aimed at better clarifying these findings in different conditions, regardless of physiology or pathology. Thus, we believe that there is a network of interactions that regulate inflammasome activity in order to maintain hematopoiesis, and an imbalance in this system is responsible for the development of hematological diseases.

## 5. The Role of Inflammasomes in Leukemias

### 5.1. Protumorigenic Effects

**Figure 3:**
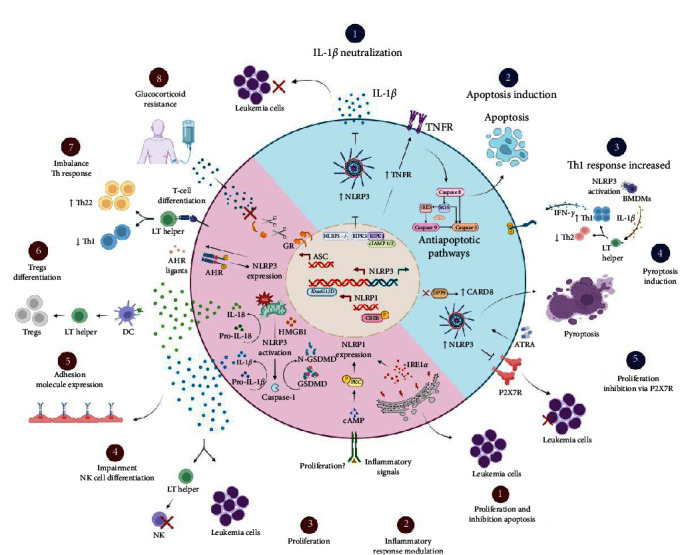
Inflammasome effects on tumor progression and inhibition in leukemias. *Antitumorigenic*: (1) the deletion of NLRP3 in leukemic cells can decrease the proliferation of blasts through the neutralization of the cytokine IL-1*β*; (2) the RIPK3 protein suppresses the proliferation of leukemic cells through the activation of NLRP3 and induces apoptosis via TNFR; (3) NLRP3 activation in BMDMs via IL-1*β* secretion promotes the proliferation of IFN-*γ*-producing Th1 cells with anti-leukemic effects; (4) NLRP3 overexpression induced by ATRA treatment cause pyroptosis of leukemic cells; moreover, DPP9 inhibition enhances CARD8 activation also culminating in this cell death; and (5) NLRP3 overexpression negatively regulates the P2X7R receptor, inhibiting the proliferation of leukemic cells and inducing apoptosis. *Protumorigenic*: (1) IRE1*α* protein increases NLRP1 expression in leukemic cells and contributes to proliferation and survival; (2) the PKC-CREB pathway regulates NLRP1 expression in leukemic cells and possibly contributes to proliferation; (3) the IL-1*β* cytokine stimulates an increase in cell proliferation; and (4) prevents the differentiation of umbilical cord blood (UCB)-CD34^+^ into NK cells. In addition, it also (5) stimulates the expression of adhesion molecules to promote the recruitment of leukemic cells by epithelial cells; (6) DCs activated by the cytokine IL-18 promotes the differentiation of TCD4+ lymphocytes into Tregs; (7) NLRP3 collaborates with AHR for imbalance in Th1 profile response; (8) increased transcription of CASP1 and NLRP3 results in cleavage of glucocorticoid receptors. Abbreviations: ⊥ = inhibition; ↓ = activation; AHR, aryl hydrocarbon receptor; ASC, apoptosis-associated speck-like protein; ATRA, all-*trans*-retinoic acid; BMDMs, bone-marrow-derived macrophage; cAMP, cyclic adenosine monophosphate; CREB, cAMP-response element-binding protein; DC, dendritic cell; DPP9, dipeptidyl peptidase 9; GR, glucocorticoid receptor; GSDMD, gasdermin-D; GSDMD-N, GSDMD amino-terminal cell death domain; IFN-*γ*, gamma interferon; IL-18, interleukin-18; IL-1*β*, interleukin-1*β*; IREI*α*, endoplasmic reticulum stress sensor; LT, lymphocyte; NK, natural killer cell; NLRP1, NOD-like receptor protein 1; NLRP3, NOD-like receptor protein 3; P, phosphorylation P2X7R, purinergic P2X7 receptor; PKC, protein kinase C ROS, reactive oxygen species; tBID, proapoptotic protein; Th1, type 1 T helper (Th1); Th22, type 22 T helper (Th22); TNFR, tumor necrosis factor receptor; Tregs, regulatory T cells; ?, Influence on the known.

Although the role of the inflammasomes is well described for some types of cancer, such as colon cancer and melanoma, in leukemias, there are many knowledge gaps, and few studies describe the mechanisms of neoplastic promotion in the disease. In Figure 3, the pro- and anti-tumorigenic mechanisms that involve the components of the inflammasome in leukemias are shown. In ALL, overexpression of the NLRP3 and ASC genes is observed in diagnostic [62] and relapse samples [63]. In addition, it has been shown that, in glucocorticoid-resistant lymphoid leukemic cells, there is decreased methylation of the CASP1 promoter and NLRP3, which results in increased transcription, constitutive activation of NLRP3, and caspase-1-mediated glucocorticoid receptor cleavage [63], this being a possible mechanism of failure in the initial treatment.

Recently, a study demonstrated that mRNA expression of CASP1 is increased in AML cell lines and clinical samples, especially in relapsed AML patients. In addition, high CASP1 expression was associated with poor prognosis, and CASP1 inhibition could decrease AML blast proliferation [64]. These findings suggest that CASP1 may contribute to the development of ALL and AML and may be used as a biomarker to predict prognosis and as a therapeutic target of acute leukemia patients.

Recently, chemotherapy-induced activation of NLRP3 in primary human B-ALL cells was demonstrated for the first time. The doxorubicin treatment led to an increase in the transcription of NLRP3 and CASP1 in B-ALL cells and a consequent increase in the production of the cytokine IL-1*β* when compared to the control. This indicates that the regulation of NLRP3 in leukemic cells has a similar function to that of monocytes [65].

Regarding IL-1*β*, its role is still controversial in ALL, with a 40-fold increase in IL1B gene expression being observed in B-ALL blasts cultured with hematopoietic growth factors [34] and in MSCs from patients at diagnosis [66]; however, its low expression is associated with a lower overall survival (OS) rate and event-free survival (EFS), and it is considered a predictor of relapse [67]. The *IL1B* gene is highly polymorphic, and several single-nucleotide variations (SNV) have been associated with increased or decreased secretion of the cytokine IL-1*β* [68], which is a possible cause for the difference in expression in populations due to ethnic/geographic variations.

Expression of the *NLRP1* gene is absent in CD34^+^ blast hematopoietic cells; however, during their differentiation, it is expressed in granulocytes. Studies demonstrate that *NALP1* levels are increased in bone marrow samples from some patients with acute leukemia but not in solid tumor samples [69]. Therefore, it has been claimed that the induction of NALP1 by a PKC activator (protein kinase C) or cAMP analogs is mediated by CREB (cAMP-response-element-binding protein), a transcription factor that regulates cellular response pathways, including proliferation, survival, and differentiation. When expression of a dominant negative form of CREB is used, reduced NLRP1 expression can be observed. Therefore, the regulation of *NLRP1* by the CREB pathway in myeloid cells may contribute to modulating the response of these cells to inflammatory stimuli and favor the survival of the leukemic clone [70].

In AML, it was demonstrated in a murine model that the presence of the *KrasG12D* mutation is responsible for the activation of NLRP3 through the production of ROS via the Kras-RAC pathway. Furthermore, the deletion of the *NLRP3* gene was responsible for the reduction of AML blast proliferation and the restoration of normal hematopoiesis [71]. HMGB1, an important DAMP that is released during the inflammatory process, seems to be directly involved in the activation of NLRP3, and it contributes to the progression of AML via the HMGB1/NLRP3/IL-1*β* axis [25]. Furthermore, studies have demonstrated that IL-1*β* secretion by AML blasts can impair the differentiation of umbilical cord blood (UCB)-CD34^+^ cells into precursors of natural killer (NK) cells in an *in vitro* coculture model [72] and stimulate the expression of adhesion molecules to promote recruitment by epithelial cells [73].

In samples from patients that were newly diagnosed with AML, an increase in the expression of NLRP3 was observed when compared to the controls and the patients in remission. NLRP3 was correlated with the increase in the expression of the aryl hydrocarbon receptor (AHR). The AHR is involved in the modulation of the immune system, specifically in the differentiation of helper T lymphocytes. In this study, an imbalance of T-helper lymphocyte subpopulations with an increase in the Th22 profile and a decrease in Th1 in de novo AML patients was also observed, which leads us to think that NLRP3, together with AHR, may cooperate in the development of AML and influence the T lymphocyte differentiation [74].

In the plasma of patients that were newly diagnosed with AML, the cytokine IL-18 was found at elevated levels [26], and *IL18* and *ASC* gene expression returned to normal after patients achieved remission [74]. In an *in vivo* model, one study demonstrated that IL-18-derived dendritic cells were able to promote the differentiation of CD4^+^CD25^+^ Treg lymphocytes [75]. Concomitant to this, it is possible to hypothesize that IL-18 could facilitate the polarization of Tregs and, in TME, it could suppress the immune response and promote the development of AML.

In CML, in an *in vitro* model, *NLRP1* was expressed at high levels by K562 cells [70], and in patients with CML, it was associated with resistance to imatinib [76]. Furthermore, the IRE1*α* protein, an endoplasmic reticulum stress sensor involved in AML progression, may increase NLRP1 expression in CML. In primary cells from patients with CML, an overexpression of IRE1*α* and NLRP1 was observed that led to cell proliferation and inhibition of apoptosis. Moreover, inhibition of this pathway led to the sensitization of CML cells to imatinib-mediated apoptosis [76]. In CML, high levels of the cytokine IL-1*β* are associated with a worse prognosis and shorter survival times [77], and in CLL, IL-1*β* secretion can induce differentiation and activation of leukemic cells [78].

### 5.2. Antitumorigenic Effects

The antitumor activity of inflammasomes may vary depending on the cell type being activated and on the interactions with TME cells. In AML, NLRP3 knockout in leukemic cells *in vivo* was able to decrease blast proliferation in bone marrow, liver, and spleen by neutralizing the cytokine IL-1*β* [26]. On the other hand, Liu et al. [79] demonstrated that the activation of NLRP3 in healthy bone marrow-derived macrophages (BMDMs) promoted the differentiation of TCD4+ lymphocytes in the Th1 profile through the secretion of IL-1*β* and high levels of Th1 cells promoted apoptosis and inhibited the proliferation of leukemic cells via IFN-*γ* secretion *in vitro* and *in vivo* (Figure 3).

Together, these results show us that the regulation of NLRP3 inflammasome activity, especially with a focus on IL-1*β*, may contribute to a new therapeutic approach in AML. In the AML TME, Th1 profile lymphocytes and IFN-*γ* production are downregulated. In this way, NLRP3-activated BMDMs promote the proliferation of IFN-*γ*-producing Th1 cells with anti-leukemic effects and may provide information that will serve as a basis for immunotherapy in AML.

In addition, receptor-interacting protein kinase 3 (RIPK3) can promote the differentiation of leukemia-initiating cells (LICs) through the activation of the inflammasome. RIPK3 suppresses myeloproliferative neoplasms by activating the inflammasome, thereby promoting differentiation. Furthermore, RIPK3 downstream of TNFR1 is responsible for inducing cell death. RIPK3 activation is regulated by the ubiquitination status of RIPK1, which in turn is controlled by a cellular inhibitor of apoptosis proteins 1 and 2 (cIAP1/2). In newly diagnosed AML patients, RIPK3 expression is often reduced to prevent LICs from going into apoptosis [80]. Thus, these results indicate RIPK3 and the inflammasome as key tumor suppressors in AML.

The axis NLRP3/P2X7R seems to contribute to the apoptosis of CLL cells. The P2X7 purinergic receptor (P2X7R) is an ATP-gated ion channel that is widely expressed in HSCs and plays an important role in cancer promotion and immune system regulation. In hematopoiesis, lymphocyte growth and differentiation are modulated by P2X7R, which is overexpressed in CLL patients. In CLL, the silencing of the*NLRP3* gene is responsible for the increase in *P2X7R* expression and promotes cell growth; in contrast, overexpression of *NLRP3* induces apoptosis [81]. Therefore, the NLRP3 positive regulation seems to downregulate P2X7R, inhibit the proliferation of LLC cells, and induce apoptosis and, as such, is a promising therapeutic target for disease.

The role of pyroptosis in cancer is controversial, since it can be beneficial or harmful for anti-tumor immunity. Liu et al. [82] reported that upon incubation with CD19^+^ leukemic cells, CAR-T cells could increase the release of lactic dehydrogenase (LDH) and upregulate the expression of gasdermin-E (GSDME) and IL-1*β*. This suggests that CAR-T cells can activate GSDME-mediated pyroptosis by releasing a large amount of perforin and GzmB and could trigger antitumor immunity [82]. However, since high expression of GSDME is observed in B-ALL cells, this could induce cytokine release syndrome and may impede the application of pyroptosis-related CAR-T therapy in leukemia patients.

In AML, studies have identified the CARD8 as a novel inflammasome sensor that triggers pyroptosis myeloid leukemia cells upon inhibition of dipeptidyl-peptidases (DPP) with Val-boroPro treatment [83]. In addition, recent studies have demonstrated that DPP9 (dipeptidyl-peptidase 9) constitutes the relevant DPP restraining CARD8-induced pyroptosis in resting human lymphocytes [84] and is not restricted to myeloid cells. These data suggest that AML cells might be sensitive to DPP8/9 inhibitors but also indicates more potential for toxicity in human resting lymphocytes. Furthermore, these results reveal exciting opportunities to modulate inflammasome activation for therapeutic benefit in leukemia patients.

Ninj1 is an adhesion molecule with an essential role in the induction of the plasma rupture membrane, the subcellular event that precedes pyroptotic cell lysis [85]. This protein is overexpressed in cells of the B-ALL lineage, and it has been studied as a potential biomarker for monitoring minimal residual disease [86]. The failure of treatment in cases of leukemia is largely due to the development of drug resistance to apoptosis. Therefore, the introduction of nonapoptotic programed cell death, such as pyroptosis, may be an effective way to rechallenge the resistance to apoptosis.

Recently, a study observed that CASP1 had lower expression in patients with acute promyelocytic leukemia (APL), mainly in relapsed patients. After all-*trans*-retinoic acid (ATRA)-treatment of APL cells, it was observed an increase of CASP1 expression via IFN-*γ*/STAT1 pathway resulted in pyroptosis and differentiation of APL cells [87]. Thus, ATRA-induced activation of CASP1 could serve as a suppressor in APL progression. Interestingly, high levels of mRNA of expression CASP1 were found in the clinical remission patient group, and CASP1 and NLRP3 expression were associated with better OS and EFS, respectively [88]. This indicates CASP1 and NLRP3 as potential biomarkers for risk stratification in ALL.

In the absence of caspase-1, NLRP3 inflammasome uses caspase-8 as both a proapoptotic initiator and a major IL-1*β*-converting protease [89]. In the presence of caspase-1, caspase-8 acts as a positive modulator of the NLRP3-dependent caspase-1 signaling cascades that drive both IL-1*β* production and pyroptotic death. In ALL, effective cell death in B- and T-ALL cells depends on the presence of caspase-8 for most cytotoxic drugs routinely used in antileukemia treatment (e.g., methotrexate) [90]. Moreover, high levels of caspase-8 protein are observed during drug-induced apoptosis of CLL cells [91, 92]. Little is known about the role of caspase-8; however, these results indicate that caspase-8 is crucial for the high anti-leukemic efficiency of numerous routine cytotoxic drugs, and it can be a promising pathway for the development of new therapeutic targets in leukemia.

### 5.3. Polymorphisms

Recent investigations have identified some SNVs that cumulatively may provide a high risk for the development of ALL [93]. Due to their multifactorial etiology, it is important to investigate the association of polymorphisms in leukemias, especially in genes of inflammatory pathways, since they may serve as potential predictors for the development and prognosis of leukemias.  Table 1 summarizes the main findings regarding polymorphisms in genes of the inflammasome complex in leukemias.

The *NLRP1* rs12150220 polymorphism causes increased *NLRP1* and IL-1*β* processing and have been associated with protection against infectious comorbidities, such as cytomegalovirus, toxoplasmosis, rubella, varicella, and parasitic diseases, in pediatric patients with ALL [94]. *IL1B* is a crucial mediator of the inflammatory response, and its role in protection from bacterial infection has previously been summarized [102]. Since patients with acute leukemia have an increased risk of developing infections, both because of leukemia and because of its treatment, further studies are necessary to evaluate the role of *NLRP1* rs12150220 in order to be able to predict the risk of infections in ALL.

Interestingly, the *NLRP3* rs35829419 and rs4353135 polymorphisms are associated with the risk of AML [95] and ALL [96] in the Asian population. These variations cause increased production of IL-1*β* and possibly contribute to chronic stress in the promotion of AML progression via an HMGB1/NLRP3/IL-1*β* dependent mechanism [25]. In addition, the P2X7/NLRP3 pathway plays an essential role in amplifying inflammation via an ATP feedback loop, thus promoting the inflammatory response; however, the function of the P2X7 receptor is not fully understood, but it is involved in ATP-induced apoptotic death in hemopoietic and CLL cells [81]. Thunberg et al. [97] demonstrated an association between *P2X7* rs3751143 with longer event-free survival in CLL patients in Sweden. In this context, a loss of P2X7 function caused by the polymorphism could be responsible for limiting CLL cell proliferation and contribute to survival. We believe that studies with a focus on the axis NLRP3/P2X7/IL-*β* could bring great discoveries about the role of the P2X7 polymorphism in CLL *in vivo*.

The *CASP8* 6N del at position-652 in the promoter region of the CASP8 gene is found to abolish the binding site for the transcriptional activator 1 (Sp1), thereby resulting in a decreased expression of the CASP8 protein in lymphocytes. Abdullah et al. [100] demonstrated that the CASP8-652 6N insertion polymorphism is associated with an increased risk of both CML and AML in the Iraqi population. These findings suggest that CASP8 6N del polymorphism might be a useful marker for determining genetic susceptibility to CML and AML.

Some studies have reported that children with mixed ancestry have a higher risk of developing ALL due to the existence of *INDEL*-type polymorphisms, which is characteristic of South American natives [103]. In the Brazilian Amazon, which has predominantly South American ancestry, the *IL1B-511 C>T* rs16944 polymorphism was associated with the risk of children developing ALL. This variation causes an increased IL-1*β* transcription and, consequently, an intense inflammasome activation. Furthermore, in ALL, it was associated with an increase in transcription of *NLRP3* and *ASC* [98] and with the risk of development of ALL in the Asian population [96]. On the other hand, this polymorphism can predict a favorable cytogenetic risk group in AML [95]. Since IL-1*β* is one of the main components of inflammasomes involved in pathogenesis in leukemias, the study of genetics can contribute to the discovery of new biomarkers that can predict clinical parameters in the disease.

Little is known about the role of the *IL18* gene in leukemia. Studies have demonstrated the link between the *IL18* rs187238 polymorphism and increased risk of CLL in the Turkish population [101], and the rs1946518 polymorphism is linked to a risk of pediatric ALL in the Asian population [96]. In addition, it was associated with increased IL-18 secretion in ALL in an Asian population [98]. CARD8 rs2041132 is important for the regulation of inflammasome activity and was associated with the risk of development of ALL in the Asian population [98]. However, further investigations are necessary to understand the role of *CARD8* polymorphisms in ALL since it is rarely described in the literature.

Collectively, these findings indicate a potential role of the genetics of inflammasomes as predictors of the development of leukemia. However, since ethnicity has a direct influence on the incidence of leukemias [104], we believe that these findings should be validated in various populations with large sample sizes in order to better understand its role in leukemias.

## 6. Inflammasome as a Therapeutic Target in Leukemia

Inflammasomes and immune response pathways have opened up avenues for several exciting new drug targets for leukemia. As inflammasomes appear to play a role in leukemia originating from multiple genetic defects, promising outcomes are expected from its drug targets.  Table 2 shows the recently discovered potential therapeutic agents for targeting the inflammasome pathway in leukemias.

Upregulation of the plasma membrane receptor ILIRAP in AML patients has been reported and can distinguish biological characteristics of leukemic clones from normal progenitors. In this context, IL1RAP blocking seems to be a good therapeutic approach in AML. In this way, the blocking of the IL1-IL1RAP signaling axis has been associated with the reduction of inflammation in the bone marrow niche and thereby promotes normal hematopoietic recovery from AML proliferation after chemotherapy [105]. Based on this, an immuno-therapy targeting IL1RAP in AML-relapsed patients has been tested using chimeric antigen receptor T-cells immunotherapy to validate this approach in the treatment of the disease (NCT04169022).

In addition, the intracellular IL-1*α* is a chromatin-associated cytokine and can affect transcription through the activation of NF-*κ*B and specific protein 1 (SP1). Studies have demonstrated a spontaneous expression of IL-1*α* in ALL blasts [106, 107]. The inhibition of IL-1*α* has been studied in aneurysmal subarachnoid hemorrhage to reduce the inflammation process and can be an alternative target for the treatment of hematologic malignancies, especially leukemias (NCT03249207).

Bruton tyrosine kinase (BTK) regulates NLRP3 inflammasome activity via direct interaction with ASC and NLRP3. Ibrutinib, a BTK inhibitor, prevents the formation of ASC specks and caspase-1 activation. It was observed that the antileukemia actions of ibrutinib demonstrated profound and immediate inhibition of CLL cell proliferation and promotion of high rates of CLL cell death [108]. In addition, combined therapies comprising ibrutinib and anti-CD19 CAR-T cells in patients with CLL after ibrutinib failure are considered feasible and safe and have already been studied (NCT05020392).

Recently, a phase I clinical trial was initiated to evaluate the best dose, possible benefits, and side effects of interleukin-18-secreting autologous anti-CD19 CAR T-Cells (huCART19-IL18) in treating patients with non-Hodgkin's lymphoma (NHL), CLL, and ALL. huCART19-IL18 targets CD19^+^ cells in NHL, CLL, and ALL and produces the cytokine IL-18. This new approach presents a potential treatment for new or relapsed patients. Finally, we believe that understanding the inflammasome pathway will be crucial for further identification of novel and improved therapeutic outcomes against leukemias.

## 7. Future Perspectives

In recent years, there has been an increase in studies involving inflammasomes in hematological malignancies, which have the intention of understanding the dual effects that these complexes exert on the physiological and malignant regulation of hematopoiesis and can thereby reveal potential clinical implications of the inflammasome. Here, we list some potential therapeutic options through the targeted modulation/inhibition of specific inflammasomes that may help maintain/restore adequate hematopoietic homeostasis.

Hu et al. [56] demonstrated that mice that were deficient in the double-stranded DNA sensor AIM2 are protected from radiation, thus indicating that DNA damage caused by radiation mediates the AIM2 inflammasome activation and cell death. AIM2 mediates the caspase-1-dependent death in response to double-strand DNA breaks caused by ionizing radiation and chemotherapeutic agents. The results suggest that AIM2 may be a new therapeutic target for ionizing radiation exposure in the prevention of radiation-induced injuries, such as those that occur in radiotherapy, for example. In addition, DNA damage upregulated NLRP12 in HSCs of mice deficient in the *Fanca* DNA repair gene and contributed to improving HSC function in both mouse and human models of DNA repair deficiency and aging [60, 109]. Combined therapies using AIM2 and NLRP12 can contribute to the repopulation of HSCs and prevent cell death after radiation exposure and are a potential approach in transplant study models.

Using several preclinical tumor models, activation of CD8^+^ T cells in response to programed cell death 1 (PD-1) in tumor tissues decreases the antitumor immune response. Genetic and pharmacological inhibition of NLRP3 suppressed PMN-MDSC tumor infiltration and significantly enhanced the efficacy of immunotherapy with anti-PD-1 antibodies. This pathway, therefore, represents an intrinsic tumor mechanism of adaptive resistance to anti-PD-1 checkpoint inhibitor immunotherapy for use in leukemia treatment [110, 111].

In addition, NLRP3 inflammasome signaling regulates the targeting, engraftment, and trafficking of HSPCs and may be useful in transplantation to improve homing of HSPCs [112, 113]. Furthermore, NLRP3 also plays a critical role in the initiation of graft versus host disease (GvHD) through the secretion of the proinflammatory cytokine IL-1*β*. Studies have demonstrated that the decrease in NLRP3 expression was responsible for attenuating GvHD in a murine model, which resulted in increased graft survival [114, 115]. These findings highlight the possibility of targeting the NLRP3 inflammasome to minimize GvHD.

## 8. Conclusion

In this literature review, we summarized the current knowledge regarding genetic and molecular mechanisms of the inflammasome in leukemia development and proposed a novel view of possible contributions and mechanisms of the inflammasome in the disease. Finally, with several therapies targeting the inflammasome currently in clinical development, we hope this study can enhance our understanding of the complexity of the relationship between inflammation and leukemogenesis and will serve as a basis for promising studies in the onco-hematology field.

## Figures and Tables

**Table 1:** Functional effect of single-nucleotide variants (SNVs) on the genes of the inflammasome complex in leukemias.

Gene	Variation	rs	Effect	Key findings	Ref.
*NLRP1*	c.464 A>T p. L155H	rs12150220	Increased IL-1ß processing; increased *NLRP1* expression	Protection against infectious diseases in pediatric patients from the Brazilian Amazon with ALL	[94]

*NLRP3*	c.2113 C>A p. Q705K	rs35829419	Increased production of IL-1ß and IL-18	Risk of AML in the Asian population	[95]
	N/A	rs4353135		Risk of ALL in the Asian population	[96]

*P2X7*	A>C p. Glu496Ala	rs3751143	Loss of function	Longer event-free survival in patients from Sweden with CLL	[97]

*CARD8*	30T>A p. C10X	rs2043211	Increased NLRP3, CASP1 and IL-1*β* transcription	Risk of ALL in the Asian population and T-cell immunophenotype	[98]

*CASP8*	−652 6N ins/del −/CTTACT	rs3834129	Decreased production of Caspase-8	Risk of CML in the Asian population	[99]
				Risk of CML and AML in the Iraqi population	[100]

*IL1B*	c.−*511* C>T	rs16944	Increased IL-1*β* transcription	Risk of developing ALL in children from the Brazilian Amazon	[94]
				Increased transcription of *NLRP3* and *ASC* in ALL in the Asian population	[98]
				Favorable-risk cytogenetics group in AML	[95]
				Increased risk of ALL in Asian children	[96]

*IL18*	c.−*137* G>C	rs187238	Increased IL-18 transcription	Risk of CLL in the Turkish population	[101]
	c.−*607* C>A	rs1946518		Increased IL-18 secretion in ALL in an Asian population	[98]
				Risk of pediatric ALL in the Asian population	[96]

Abbreviations: p, protein change; c, changed allele; rs, reference sequence; ALL, acute lymphoblastic leukemia; AML, acute myeloid leukemia; CLL, chronic lymphoid leukemia; CML, chronic myeloid leukemia; Ref, references.

**Table 2:** Potential therapeutic targets directed to the inflammasome.

Therapeutic potential	Description	Target	Phase	Ref./NTC number
IL1RAP receptor inhibition	Despite progress, there is still a need to provide additional strategies for patients with refractory AML. This study aims to evaluate the effects of immunotherapy for refractory AML cells expressing IL1RAP using chimeric antigen receptor T cells	IL1RAP	Phase I	NCT04169022

IL-1R*α* receptor inhibition	This study aims to evaluate the effects of subcutaneous administration of an IL-1*β* receptor antagonist on the clinical status of patients with aneurysmal subarachnoid hemorrhage	IL-1R*α*	Phase III	NCT03249207

BTK inhibitor	Anti-CD19 chimeric antigen receptor (CAR) T-cell has shown dramatic efficacy in B-cell malignancies, and Bruton tyrosine kinase (BTK) inhibitor agents have been validated as an effective drug to treat B-cell malignancies. Thus, combined therapies in patients with CLL after ibrutinib failure are considered feasible and safe	CD19^+^ cells	Phase III	NCT05020392

huCART19-IL18	Interleukin-18 secreting autologous anti-CD19 CAR T-cells (huCART19-IL18) target CD19 cells in NHL and CLL and produce IL-18. This approach can be used by both new and relapsed patients	CD19^+^ cells	Phase I	NCT04684563

Abbreviations: IL1RAP, interleukin 1 receptor accessory protein; AML, chronic myeloblastic leukemia; BTK, Bruton tyrosine kinase, IL-1R*α*, interleukin 1 receptor type I; CLL, chronic lymphocytic leukemia; CART-T, chimeric antigen receptor (CAR) T-cell.

## Data Availability

Data sharing is not applicable to this article, as no new data were created or analyzed in this study.
